# Cloacal Bacterial Diversity Increases with Multiple Mates: Evidence of Sexual Transmission in Female Common Lizards

**DOI:** 10.1371/journal.pone.0022339

**Published:** 2011-07-21

**Authors:** Joël White, Murielle Richard, Manuel Massot, Sandrine Meylan

**Affiliations:** 1 Ecologie & Evolution (UMPC-ENS-CNRS), Université Pierre et Marie Curie, Paris, France; 2 Evolutionary Ecology Group, University of Antwerp, Antwerp, Belgium; 3 Institut Universitaire de Formation des Maîtres de Paris, Ecole interne de l'Université Sorbonne–Paris IV, Paris, France; Martin-Luther-Universität Halle, Germany

## Abstract

Sexually transmitted diseases have often been suggested as a potential cost of multiple mating and as playing a major role in the evolution of mating systems. Yet there is little empirical data relating mating strategies to sexually transmitted microorganisms in wild populations. We investigated whether mating behaviour influences the diversity and composition of cloacal assemblages by comparing bacterial communities in the cloaca of monandrous and polyandrous female common lizards *Zootoca vivipara* sampled after the mating period. We found that polyandrous females harboured more diverse communities and differed more in community composition than did monandrous females. Furthermore, cloacal diversity and variability were found to decrease with age in polyandrous females. Our results suggest that the higher bacterial diversity found in polyandrous females is due to the sexual transmission of bacteria by multiple mates. The impact of mating behaviour on the cloacal microbiota may have fitness consequences for females and may comprise a selective pressure shaping the evolution of mating systems.

## Introduction

Multiple mating by females has been the focus of numerous theoretical and empirical studies for many years [1]–[4]. All underline the importance of identifying the factors influencing such behaviour. The potential benefits of polyandry for females may be direct, such as nuptial gifts or paternal care, or indirect, arising from an increase in genetic quality or diversity of the offspring [2]. Among the many suggested costs of polyandry, which include predation, energy loss, and harmful paternity assurance tactics [5], sexual transmission of pathogens is predominantly cited and indeed has the potential for substantially reducing the benefits of multiple mating. Yet despite a relative abundance of theoretical literature on the role of sexually transmitted diseases in shaping the evolution of mating systems [6]–[8], there is little empirical data relating mating strategies to sexually transmitted microorganisms in wild populations (but see [9]).

Here, we investigate whether mating behaviour influences the diversity and composition of cloacal assemblages by examining bacterial communities in the cloaca of female common lizards *Zootoca vivipara* sampled after the mating period. Common lizards comprise a good model species to address this issue for two main reasons. First, as in many squamates, copulation duration is prolonged (up to several hours [10]) providing ample potential for bacteria present in the male reproductive tract to be transmitted to the female cloaca. Second, two alternative female mating strategies, i.e. monandry and polyandry, coexist in this species [11].

Based on the assumptions that different males likely harbour different bacterial strains (individual microbial signature [12]) and that at least some will be sexually transmitted and get established in the female cloaca [13], [14], we predict bacterial diversity in the cloaca to be higher in polyandrous females than in monandrous females at the end of the mating period. Further, we expect multiple mating to lead to greater variability in cloacal community composition among polyandrous females than among monandrous females.

## Materials and Methods

### Ethics statement

The study was carried out in compliance with French law, with agreement of the Parc National des Cévennes and the French ministry of science and education, and received approval by the French governmental Veterinary Services (permit number # 77–02).

### Study population and data collection

The common lizard is a small non-territorial lacertid, widely distributed throughout Eurasia. Our study population is located in Mont-Lozère, south-eastern France (44°30′N, 3°45′ E) and has been used in a demographic study over the last 20 years [15]. In this population, males emerge from hibernation in mid-April, followed by females in mid-May, at which time mating starts. Females mate with one or several males, some or all of whom may sire the offspring [16]. Here we define polyandry as multiple males contributing to the litter.

In mid-June 2009, 38 gravid females were captured, measured (snout-vent length in mm, hereafter referred to as body size), weighed (to the nearest 0.005 g) and identified, thus providing their minimum age. Identification is based on toe clipping, which has been shown to have no effect on subsequent recapture and survival probabilities [17]. On the day of capture, the cloacal bacterial assemblage of each female was sampled by flushing the cloaca with 50 µl of a sterile saline solution (Phosphate buffer saline, pH 7.4, Sigma) using a sterile pipette. The sample was immediately placed in a sterile 1.5 ml vial and stored at −20°C. Each female was sampled twice to ensure maximum bacterial collection. Duplicate samples were highly repeatable (see Text S1) and were therefore used jointly in further analyses. Before each sampling, the exterior of the cloaca was cleaned with alcohol to avoid contamination from bacteria outside the cloaca.

### ARISA and analyses of bacterial communities

To characterize the structure and the diversity of bacterial communities present in each cloaca sample, we performed automated ribosomal intergenic spacer analyses (ARISA, [18]). This DNA-fingerprinting method is based on the amplification of the internal transcribed spacer (ITS) region lying between the 16S and 23S rRNA genes in the ribosomal operon. The ITS region is extremely variable in both sequence and length for different prokaryotic species, thus the DNA amplification profile obtained with ARISA allows straightforward estimation of bacterial diversity, avoiding biases inherent of classical culture-based techniques. The details of DNA extraction, PCR amplification and genotyping are provided in White et al. [13]. For each cloacal sample, the sequencer produced an ARISA profile in which each peak corresponds to one phylotype, or operational taxonomic unit (OTU). In the various cloacal samples, the sequencer detected ITS fragments ranging from 315 to 1189 base pairs in size, representing a cumulated total of 67 OTUs.

The structure and diversity of the bacterial communities were analysed with PRIMER v6.1 [19]. The ARISA profiles were all standardised (by total abundance, [19]) to correct for any bias due to differences in the quantity of DNA extracted and amplification efficiency. Cloacal assemblage diversity was calculated using the Simpson diversity index (1-λ', [19]). The relative similarities of cloacal bacterial assemblages among females were graphically analyzed using a non-metric multidimensional scaling (nMDS) based on a zero-adjusted Bray-Curtis similarity matrix (see [19] for details). The reliability of the nMDS representation of the assemblage similarities was assessed with its stress values; stress <0.2 is usually considered to be acceptable [20]. Variability among cloacal assemblages according to female mating strategy and/or age was examined using relative multivariate dispersion values [20].

### Assessment of female polyandry

All females were housed in terraria until parturition using a standardized protocol [15]. Female polyandry was assessed by carrying out microsatellite marker-based paternity analyses within litters. Genomic DNA of females and juveniles was extracted from tail-tips using the QIAquick96 Purification Kit (Qiagen). Individuals were genotyped using eight microsatellite markers, 6 routinely used [11] and two new ones (Genebank A.N.: HM191619 and HM191620). None of the loci deviated from Hardy-Weinberg equilibrium. Multiple paternity was inferred from the genotypes of juveniles after the subtraction of maternal alleles and by investigation of juvenile genetic similarity. We used a binary variable, i.e. singly vs. multiply sired litters.

### Statistical analyses

We found strong correlations between female age, body size and body mass at capture (R between 0.54 and 0.87, P<0.0002) preventing us from including them as simultaneous covariates in the model. The respective effects of age and body size on cloacal bacterial diversity were therefore tested separately and we tested for body condition, calculated as the residuals of the regression between body mass and size. All statistical analyses were performed with a generalised linear model (MIXED procedure, SAS Institute Inc., 9.2), selecting final models after backward elimination of independent factors with P>0.05 and checking residuals of the initial models for normality and homoscedascity.

## Results

**Figure 1:**
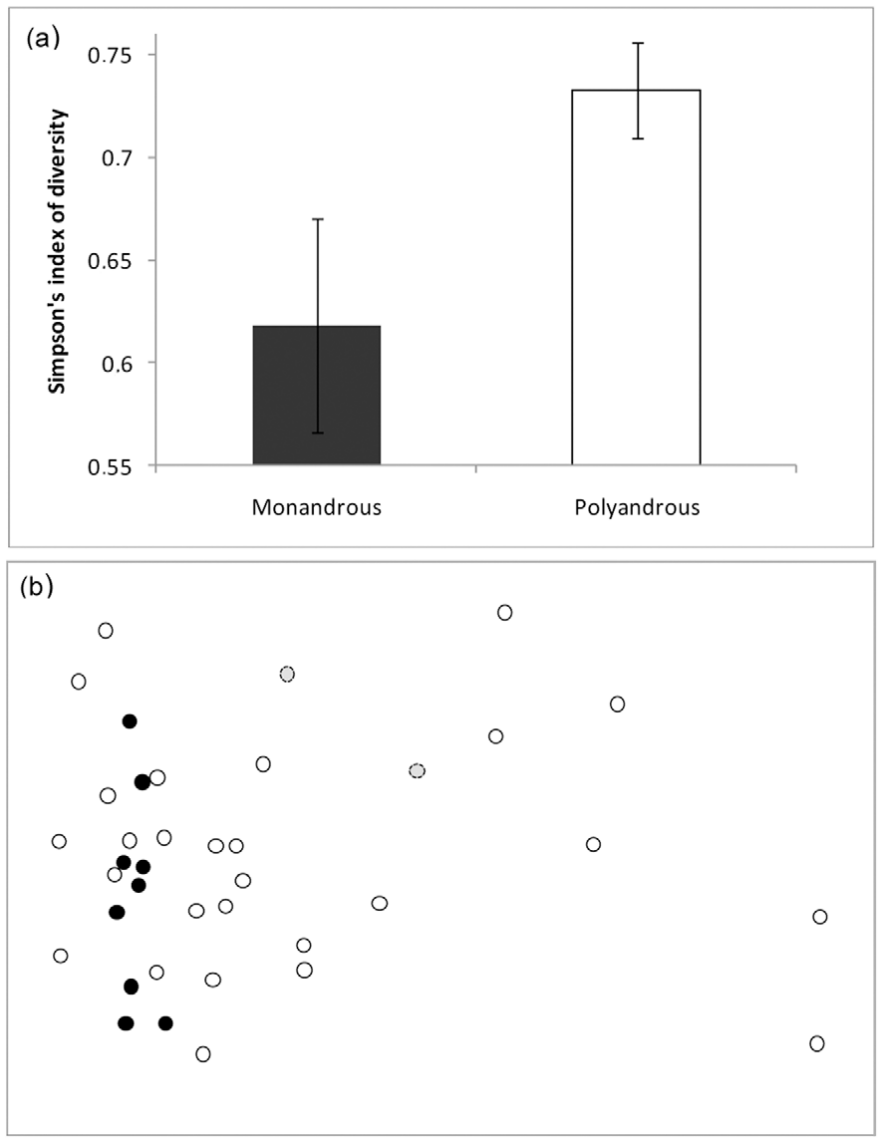
Differences in bacterial communities. Differences in bacterial communities in the cloaca of monandrous (n = 11) and polyandrous (n = 27) females in terms of (a) diversity (Simpson's index, errors bars represent SE) and (b) composition represented by nMDS ordination (2D stress: 0.16; full circles: monandrous, grey circles: monandrous graphical outliers, empty circles: polyandrous).

We investigated the relationship between bacterial diversity in the cloaca of female lizards and several characteristics of these females. We found that cloacal bacterial diversity was not significantly related to body size (F_1,37_ = 1.60, p = 0.21), body condition (F_1,37_ = 0.25, p = 0.62), date of capture (F_1,34_ = 1.45, p = 0.24) or age (F_1,34_ = 3.31, p = 0.08). Cloacal bacterial diversity was however highly related to the mating strategy of females, diversity being significantly higher in polyandrous females than in monandrous females (F_1,33_ = 8.29, p = 0.007; Figure 1a).

The relative similarities of the cloacal bacterial assemblages in both types of females were then plotted into the best two-dimensional nMDS ordination (2D stress: 0.16; Figure 1b). This analysis showed that cloacal assemblages were far more variable in terms of bacterial community composition in polyandrous females (relative dispersion, 1.06) than in monandrous females (relative dispersion: 0.72, without outliers: 0.46), the latter being more tightly clustered (Figure 1b).

**Figure 2:**
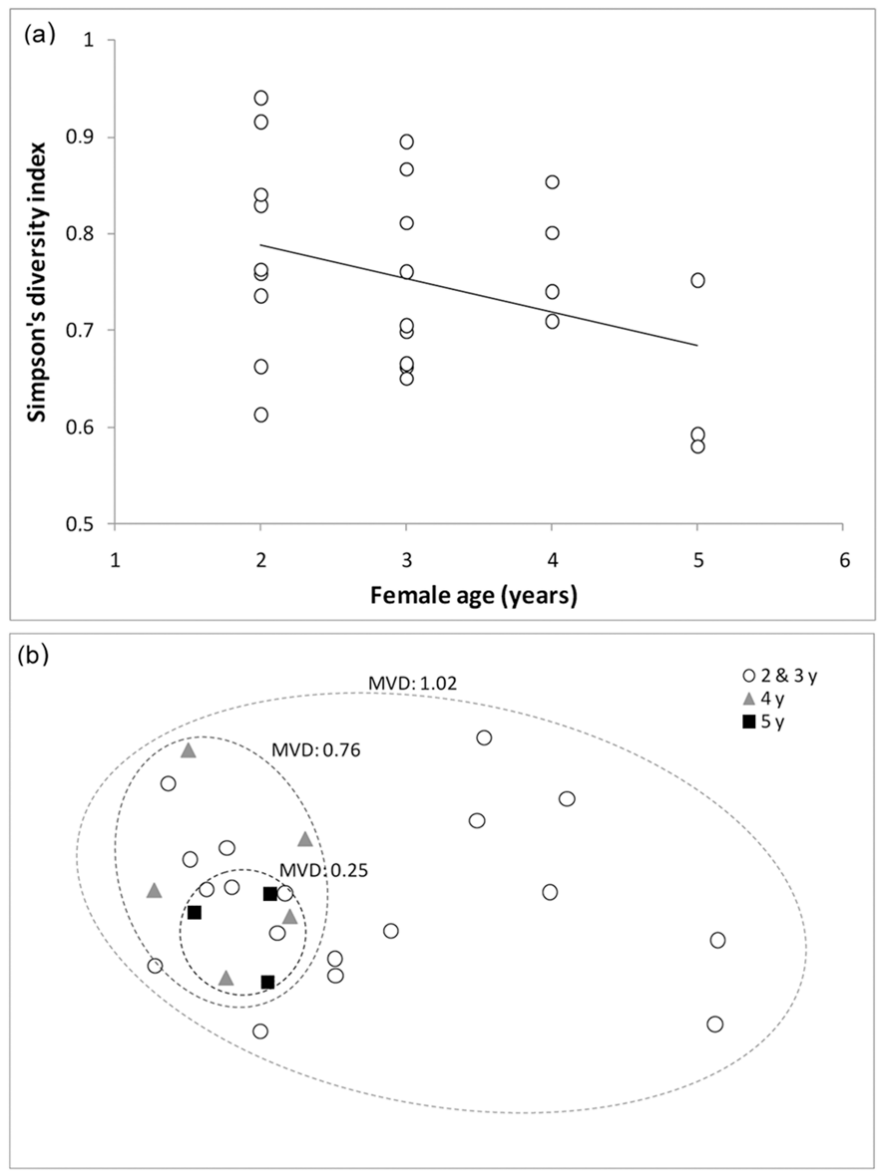
Differences in bacterial communities in the cloaca of polyandrous females according to age. (a) Simpson's diversity index according to female age (years). (b) nMDS ordination (2D stress: 0.17) of bacterial communities in females of 2 and 3 years (empty circles), 4 years (grey triangles) and 5 years (full squares). Multivariate dispersion values (MVD) for each age are represented on the graph.

As female age had a marginal effect on bacterial diversity, we also considered its effect within each mating strategy group. In polyandrous females there was a significant negative relationship between female age and bacterial diversity in the cloaca (F_1,22_ = 5.74, p = 0.03; Figure 2a) whereas no such link was found in monandrous females (F_1,9_ = 0.04, p = 0.84; age*mating strategy interaction: F_1,32_ = 0.19, p = 0.67). Furthermore, 2D nMDS ordination revealed decreasing variability in bacterial community composition with age (relative dispersion: ≤3y∶1.02; 4y∶0.76; 5y∶0.25; Figure 2b). For both mating strategies and female age, the decrease in relative dispersion was not an artefact due to the decrease in sample size (Text S2, Figure S1).

## Discussion

If bacteria are sexually transferred during copulation and different males harbour different strains, polyandrous females are expected to host more diverse bacterial communities in their cloaca after the mating period than monandrous females. In this study, multiply mated females were found to harbour a more diverse cloacal microbiota than did singly sired females. Furthermore the bacterial communities of polyandrous females were more varied in composition than that of monandrous females. Our results thus support the hypothesis that female mating strategies affect cloacal assemblages via the sexual transmission of bacteria by single or multiple mates. One cannot exclude the possibility that the differences observed have no direct link to female mating behaviour but is a consequence of physiological or health status differences between the two groups of females, which might influence the cloacal microbiota. If that were the case, one would expect bacterial diversity to be linked to relevant measures of health and fitness in our study species. In common lizards, body condition has been found to be positively related to endurance [21], [22], capacity for dispersal [15] reproductive success [23] and survival [24], [25], and to decrease with oxidative damage [26] and higher levels of stress hormones [27]. However, we found no relation between bacterial diversity and female body condition. Furthermore, we found no evidence of differences in body condition according to mating strategy (GLM, F_1,35_ = 1.38, p = 0.25) nor are we aware of any study reporting such differences in this species. In that context, differences in female condition seem to be an unlikely explanation for our findings. The higher diversity of bacteria and especially the greater variability in microbial composition in polyandrous females may thus best be explained as the result of the sexual transmission of different bacterial strains by multiple mates [9].

The low cloacal diversity and high similarity in monandrous females hints at two facts: first, female pre-mating cloacal diversity and variability may be naturally low, which confirms the findings of Martin *et al.* [12] in striped plateau lizards *Sceloporus virgatus*, and second, one (or few) copulation(s) does not seem to have much impact on female cloacal communities. An alternative explanation to the latter may be that monandrous females are choosier [28] and more able to select healthy males which are less likely to transmit invasive and/or numerous bacteria (parasite-mediated sexual selection, [29]).

An intriguing result is the decrease in cloacal bacterial diversity and variability with age in polyandrous females. It has been shown in black-legged kittiwakes *Rissa tridactyla*, that female cloacal communities are quite resilient, rapidly returning to their initial state after the increase in diversity caused by sexually transmission of bacteria [13]. In the present context, it is possible that cloacal resilience increases with age, with indigenous cloacal communities and the host immune system being increasingly efficient at resisting colonisation by foreign bacterial strains. Alternatively, this pattern may indicate a selection with age process, whereby females with higher intrinsic cloacal resilience for instance, may have a longer life expectancy.

The sexual transfer of bacteria in the gastrointestinal tract is likely to have a number of fitness consequences for females, which may be negative or positive. The addition of new bacterial species may disrupt the indigenous microbiota [30], increase the probability of pathogenic bacteria becoming established in the cloaca [31] and altogether affect female health. Alternatively, sexual transmission may provide females with the opportunity to gain beneficial bacteria [32] that produce bacteriocins lethal to resident pathogenic strains or that help in the synthesis and absorption of nutrients [33] thus providing the female with new probiotic and?or antibiotic bacteria.

To conclude, our study provides some of the first evidence suggesting that female mating strategies affect cloacal bacterial communities, potentially impacting female fitness. Our findings may shed new light on our understanding of the processes shaping the evolution of mating behaviour in promiscuous species and we hope they will stimulate further research, in particular experiments, on the role of sexually transmitted bacteria in reproductive behaviour.

## Supporting Information

Figure S1 **Comparison of multivariate dispersion values generated by random selection of subgroups (boxplots) with actual multivariate dispersion values of each subgroup (full circles).** Boxes represent 25^th^ to 75^th^ percentile, with lines being the median and whiskers the 5^th^ and 95^th^ percentile.
(TIF)

Text S1 **Bacterial sample repeatability.**
(DOC)

Text S2 **Multivariate dispersion value in relation to sample size.**
(DOC)
