# Supplementary figures and images for: Cloacal Bacterial Diversity Increases with Multiple Mates: Evidence of Sexual Transmission in Female Common Lizards
Source: PLoS One. 2011 Jul 21;6(7):e22339. doi: 10.1371/journal.pone.0022339 (PMC3141023; doi:10.1371/journal.pone.0022339)

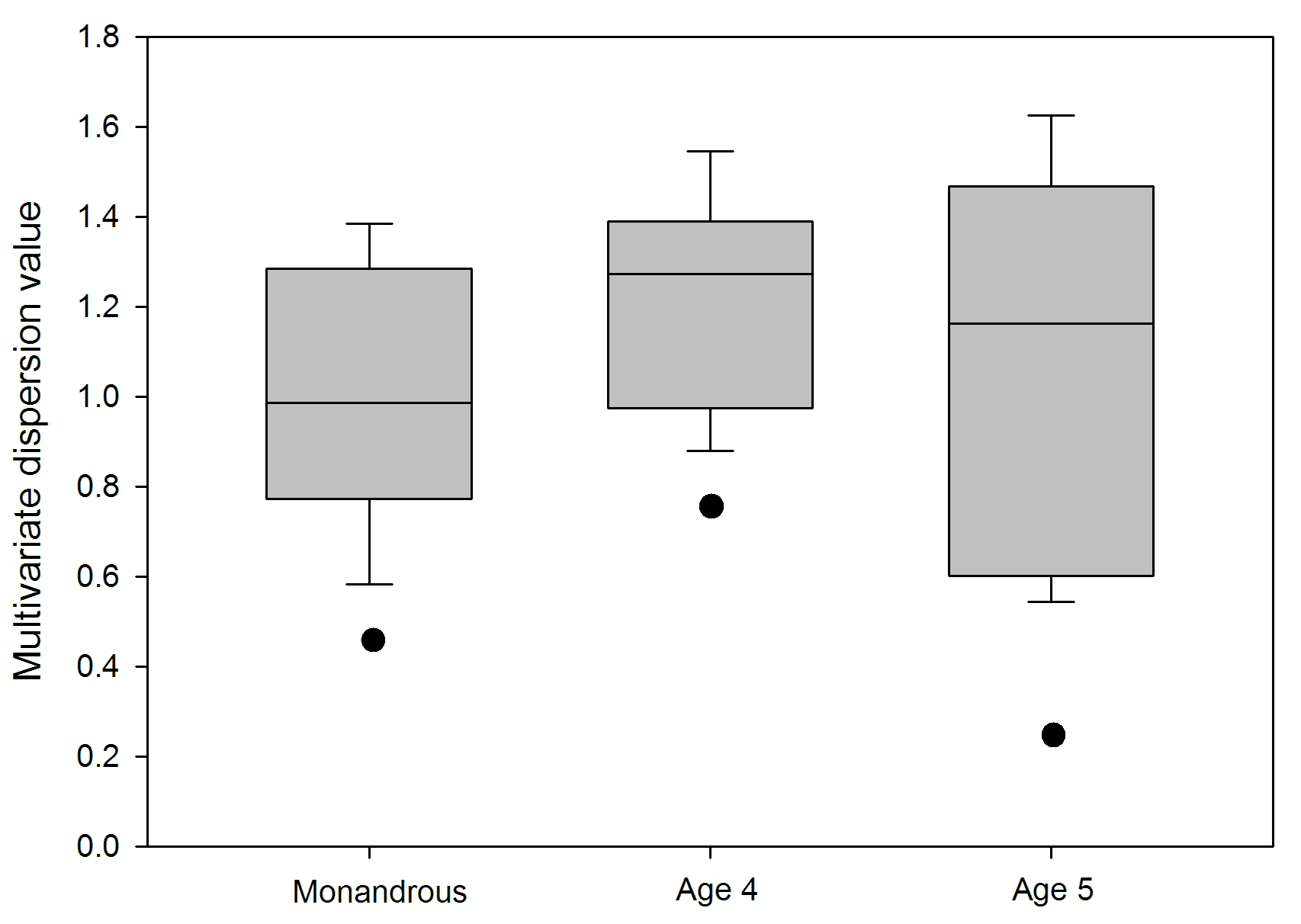

Supplement: Figure S1 — Comparison of multivariate dispersion values generated by random selection of subgroups (boxplots) with actual multivariate dispersion values of each subgroup (full circles). Boxes represent 25th to 75th percentile, with lines being the median and whiskers the 5th and 95th percentile. (TIF) [file pone.0022339.s001.tif]
