# Supplementary material for: Cloacal Bacterial Diversity Increases with Multiple Mates: Evidence of Sexual Transmission in Female Common Lizards
Source: PLoS One. 2011 Jul 21;6(7):e22339. doi: 10.1371/journal.pone.0022339 (PMC3141023; doi:10.1371/journal.pone.0022339)
Supplement: Text S1 — Bacterial sample repeatability. (DOC) [file pone.0022339.s002.doc]

**Text S1. Bacterial sample repeatability**

We compared the duplicate samples taken from each female. We found that the bacterial communities in duplicate samples were highly similar in composition (mean Bray-Curtis coefficient: 71.36±2.18%) and diversity (Pearson correlation coefficient, r=0.66, p<0.0001, n=106), underlining the repeatability of the ARISA method and allowing accurate interindividual comparisons of bacterial communities. Consequently, for all analyses, we used only one ARISA profile per female by averaging the values of the duplicate samples
