# Supplementary material for: Cloacal Bacterial Diversity Increases with Multiple Mates: Evidence of Sexual Transmission in Female Common Lizards
Source: PLoS One. 2011 Jul 21;6(7):e22339. doi: 10.1371/journal.pone.0022339 (PMC3141023; doi:10.1371/journal.pone.0022339)
Supplement: Text S2 — Multivariate dispersion value in relation to sample size. (DOC) [file pone.0022339.s003.doc]

**Text S2. Multivariate dispersion value in relation to sample size.**

In order to examine whether the reduced cloacal community variability (measured by multivariate dispersion value, MVD) found in monandrous and older polyandrous females was an artifact due to smaller sample sizes, we performed a simple bootstrap analysis. This was carried out by arbitrarily assigning a number (from 1 to 38) to the cloacal community of each female - i.e. each data point on the nMDS ordination plots (Figures1b and 2b). Using a random integer generator (<http://www.random.org/integers/>), we then randomly selected 9 data points and calculated their MVD. This process was repeated 10 times, giving 10 MVD values with a mean of 0.99±0.09. These values were compared with the MVD of the 9 actual monogamous female communities in a boxplot (Figure S1). The same bootstrap analysis was performed randomly selecting 5 data points (for age 4 polyandrous females) and 3 data points (for age 5 polyandrous females), the results of which were also compared with the actual MVD in Figure S1.

For all three subgroups (monandrous females, age 4 and age 5 polyandrous females), we found that the actual MVD values were significantly lower than expected from random sampling, indicating that the lower variability in these groups was unlikely to be due to the smaller sample sizes of these groups.
